# Fat source–dependent effects of lysophospholipid and inulin supplementation in broilers: Impacts on performance, muscle fatty acids, digestibility, enzyme activity, and intestinal morphology

**DOI:** 10.1016/j.psj.2026.106531

**Published:** 2026-01-28

**Authors:** Mozafar Rahimpour, Kamran Taherpour, Hossein Ali Ghasemi, Hassan Shirzadi

**Affiliations:** a Department of Animal Science, Faculty of Agriculture, Ilam University, Ilam, Iran; b Department of Animal Science, Faculty of Agriculture and Environment, Arak University, 38156-8-8349 Arak, Iran

**Keywords:** Broiler chickens, Digestive function, Fatty acid profile, Lysophospholipid blend, Inulin

## Abstract

Optimizing the use of dietary emulsifiers and prebiotics in relation to fat source may enhance nutrient-utilization efficiency in broiler production. This 42-d study evaluated the effects of an emulsifier (lysophospholipid; LPL) and inulin supplementation in diets with two fat sources on growth performance, nutrient digestibility, muscle fatty acid composition, digestive enzyme activity, and intestinal histomorphology. Eight hundred 1-d-old male broiler chickens were assigned to eight treatments in a 2 × 2 × 2 factorial design with two fat sources (soybean oil or beef tallow), two LPL levels (0 or 1 g/kg), and two inulin levels (0 or 1 g/kg). Interactive effects were detected for fat source × inulin on average daily gain (ADG), mortality, and the European Production Index (EPI), with inulin addition to soybean-oil–based diets yielding superior growth and survival rates (*P* < 0.05). The LPL × inulin interaction increased feed intake and improved gain-to-feed ratio (*P* < 0.05). The fat source × LPL interaction significantly influenced lipid-metabolism–related traits (*P* < 0.05); in tallow-based diets, LPL supplementation increased fat digestibility and AMEn, reduced breast fat deposition, and improved the fatty acid profile of thigh muscle by elevating n-3 PUFA and lowering the n-6/n-3 ratio, whereas no significant effects occurred in soybean-oil–based diets. When interaction terms were not significant, LPL increased protease and lipase activities and improved duodenal villus height and surface area, whereas inulin increased protease activity, improved protein digestibility, and enhanced jejunal villus architecture (*P* < 0.05). In conclusion, LPL is particularly beneficial in tallow-based diets by enhancing lipid digestibility, energy utilization, and the thigh-muscle fatty acid profile, while inulin improves growth performance, especially in soybean-oil–based diets—offering a practical strategy to optimize broiler production.

## Introduction

In modern poultry production, the dietary fat source plays a central role in determining the efficiency of energy utilization, nutrient digestibility, and product quality. Fats and oils serve not only as dense sources of energy but also influence feed palatability, gastrointestinal function, and tissue lipid deposition (Baião and Lara, 2005; Ferrini et al., 2008). Among common lipid sources, soybean oil is rich in polyunsaturated fatty acids (PUFA), particularly linoleic acid (Arbabi-Motlagh et al., 2022), whereas tallow is predominantly composed of saturated fatty acids (SFA) and monounsaturated fatty acids (MUFA) (Kanakri et al., 2018). The degree of saturation, chain length, and positional distribution of fatty acids affect micelle formation, lipid emulsification, and absorption in the small intestine, ultimately influencing the availability of energy and essential fatty acids to the bird (Carmona et al., 2017; Shoaib et al., 2023). The choice between vegetable oils and animal fats is influenced by multiple factors, with animal fats such as tallow often being more cost-effective, because they are rendering by-products of the meat industry that contribute to the circular economy and waste reduction (Wongsuthavas et al., 2011; Wickramasuriya et al., 2020), while also offering higher oxidative stability than PUFA-rich oils, thereby reducing the risk of lipid peroxidation during feed storage (Khajali and Fahimi, 2010; Pereira et al., 2017). However, the physicochemical properties of tallow, namely, its higher melting point and lower degree of unsaturation, can reduce digestibility, particularly in young birds with immature secretion of bile salts and pancreatic lipase (Okur, 2020; Poorghasemi et al., 2024). By contrast, unsaturated vegetable oils, such as soybean oil, form micelles more readily, enhancing lipid emulsification and absorption (Majdolhosseini et al., 2019; Rodríguez-Sánchez et al., 2019). The difference in digestibility between SFA-rich and PUFA-rich fats highlights the need for dietary strategies to improve the utilization of animal fats.

One approach to improving the utilization of less digestible fats is the inclusion of functional feed additives that modulate lipid digestion and absorption. Among these, lysophospholipids (LPL), derived from partial hydrolysis of phospholipids, have gained attention as potent natural emulsifiers and absorption enhancers in monogastric species (Siyal et al., 2017; Shahid et al., 2021). Owing to their amphipathic nature and reduced molecular size compared with intact phospholipids, LPL readily insert into micelles, reduce surface tension at the oil–water interface, and facilitate lipase access to triglycerides (Ko et al., 2023; Oketch et al., 2023). In poultry, supplementation with LPL has been shown to increase apparent metabolizable energy, improve ileal digestibility of fat and amino acids, and improve performance, particularly in SFA-rich diets (Xiang et al., 2024; Mohiti-Asli et al., 2025). Beyond enhancing lipid emulsification, LPL may exert broader physiological effects. They can modulate intestinal morphology by increasing villus height and surface area, enhancing nutrient transport capacity, and reducing intestinal permeability (Bassareh et al., 2023; Gholami et al., 2024). Recent studies in broilers have also reported that LPL supplementation can favorably alter meat fatty acid profiles, increase PUFA deposition, and improve feed efficiency when included in diets containing either vegetable oils or animal fats (Ahmadi-Sefat et al., 2022; Vakili and Ebrahimnezhad, 2023).

Another functional additive of interest is inulin, a nondigestible fructan classified as a prebiotic (Narala et al., 2022). Inulin escapes digestion in the upper gastrointestinal tract and is selectively fermented by beneficial microbiota such as *Bifidobacterium* and *Lactobacillus* in the ceca, leading to the production of short-chain fatty acids (SCFA) that improve gut health and immune function (Buclaw, 2017). Although fermentation occurs primarily in the ceca, SCFA and microbially mediated changes in bile acid metabolism can influence gastrointestinal function beyond the hindgut, including epithelial development and enterohepatic bile acid circulation, which directly contribute to lipid emulsification and absorption in the small intestine. In poultry, inulin supplementation has been associated with improved growth performance and enhanced intestinal morphology, including increased villus height and a higher villus height-to-crypt depth ratio (Song et al., 2018; Moreno-Mendoza et al., 2021). Importantly, inulin has also been reported to modulate lipid metabolism, potentially by reducing hepatic lipogenesis, increasing bile acid excretion, and altering fatty acid composition in muscle tissue (Kozlowska et al., 2016; Grela et al., 2021; Dunshea et al., 2024). These effects are not attributable to absorption of intact inulin in the small intestine; rather, inulin largely escapes host digestion and is fermented by cecal microbiota to SCFA that enter portal circulation and can downregulate hepatic lipogenesis (e.g., via propionate availability and SCFA-mediated signaling), while microbiota-dependent changes in bile acid deconjugation and enterohepatic cycling may increase fecal bile acid loss and modify lipid handling (Yang et al., 2023; 2024). Accordingly, inulin may influence lipid utilization through indirect pathways that interact with the lipid matrix and emulsification processes occurring proximally in the small intestine through SCFA- and bile acid–mediated effects on intestinal function and lipid absorption efficiency, rather than through direct small-intestinal uptake of inulin. While the majority of inulin research in poultry has been conducted with vegetable oil–based diets, evidence from monogastric models suggests that its lipid-modulating effects, including bile acid–binding, may be relevant across different fat sources (Chen et al., 2020; Albouery et al., 2021; Huang et al., 2023), although the extent of such interactions remains unclear.

Despite a growing body of research on LPL and inulin individually, relatively few studies have examined their interactive effects in poultry diets that differ in fat source. Because dietary fat source and LPL primarily influence emulsification and absorption in the small intestine, whereas inulin primarily acts through cecal fermentation, any combined effects are expected to arise through indirect mechanisms linking hindgut fermentation to proximal digestion (e.g., modulation of intestinal morphology and bile acid handling). Consequently, the response to LPL may depend on the fat source, and the response to inulin may vary with the lipid matrix and the degree to which emulsification is limiting. However, comprehensive evaluations of how dietary fat source interacts with LPL and inulin supplementation to concurrently influence nutrient utilization, metabolic health, and meat quality in broilers remain scarce. Therefore, this study aimed to investigate the main and interactive effects of the dietary fat source (soybean oil vs. tallow), LPL, and inulin supplementation on growth performance, metabolic profile, body composition, fatty acid composition of breast and thigh muscles, digestive enzyme activity, nutrient digestibility, and gut morphology in broiler chickens.

## Materials and methods

### Fat sources and additives

Both fat sources (soybean oil and beef tallow) were subjected to proximate and quality assessments before inclusion in the diets. According to AOCS (2011), analytical parameters included moisture content, impurities, unsaponifiable matter, free fatty acid (FFA) percentage, and peroxide value (PV). Moisture and impurities were determined gravimetrically by drying and filtration. Unsaponifiable matter was measured by hydrolyzing a known fat sample with ethanolic KOH, extracting unsaponifiable compounds into a solvent (petroleum ether), and then evaporating and weighing the residue as a percentage of the original fat. Free fatty acids were quantified by titrating the fat dissolved in a neutral solvent with sodium hydroxide to a phenolphthalein endpoint; values are expressed as % oleic acid equivalent. Peroxide value was measured using the iodometric method, with results expressed as meq O₂/kg fat. These analyses confirmed that moisture and impurities were each below 0.1% in both fats. Unsaponifiable matter was within typical ranges (approximately 1.05% for soybean oil; 0.85% for tallow). FFA levels were low (0.2% in soybean oil; 0.65% in tallow). Peroxide values were 2.8 meq O₂/kg fat for soybean oil and 4.8 meq O₂/kg fat for tallow. The fatty acid composition (relative %) of the experimental fat sources is presented in Table 1.

**Table 1:** Fatty acid composition (relative %) of the experimental fat sources.

Item^1^	Soybean oil	Beef tallow
C14:0	0.075 ± 0.002	1.95 ± 0.03
C16:0	12.8 ± 0.3	28.0 ± 0.3
C17:0	0.052 ± 0.002	1.34 ± 0.04
C18:0	4.5 ± 0.2	14.1 ± 0.5
C20:0	0.126 ± 0.005	0.89 ± 0.04
C16:1	0.095 ± 0.02	3.0 ± 0.2
C18:1	22.2 ± 0.7	45.2 ± 0.9
C20:1	0.19 ± 0.02	2.26 ± 0.1
C18:2n-6 (LA)	53.3 ± 1.2	2.85 ± 0.03
C18:3n-3 (ALA)	6.2 ± 0.2	0.093 ± 0.005

1 Mean and standard deviation of three samples.

The emulsifier was a commercial lysophospholipid premix (Artifire) included at 0.10% of the diet. According to the manufacturer’s specifications for the trial, the product contained 25% lysophospholipids (lysophosphatidylcholine, lysophosphatidic acid, lysophosphatidylinositol, lysophosphatidylethanolamine), 40% lecithin, 6% glyceryl polyethylene glycol ricinoleate (PEG-ricinoleate; E 484), and 29% carrier. The prebiotic was inulin (commercial brand Raftiline®, BENEO-Orafti S.A., Tienen, Flemish Brabant, Belgium), included at 0.10% of the diet. Raftiline denotes chicory-root inulin with a degree of polymerization (DP) typically spanning approximately 5 to 60 DP, available in grades such as HP/HPX (high-DP inulin; DP ≥ 23) and GR (granulated).

### Birds and treatments

All experimental procedures were conducted in accordance with the guidelines of the Animal Care and Use Committee of Ilam University (Ilam, Iran) and were approved under the relevant institutional protocol (contract number IU-98-189971). A total of 800 d-old male broiler chicks (Ross 308) were randomly allocated to eight dietary treatments with five replicates per treatment, resulting in 20 birds per replicate pen. Each replicate consisted of one experimental pen measuring 2.0 × 1.6 m. The experiment was arranged as a completely randomized design (CRD) with a 2 × 2 × 2 factorial treatment arrangement in a completely randomized design, with two dietary fat sources (soybean oil or beef tallow), two levels of LPL supplementation (0 or 0.10%), and two levels of inulin supplementation (0 or 0.10%). During the first three days, chicks were provided with continuous 24-h lighting; thereafter, a lighting program of 23 h of light and 1 h of darkness per d was maintained until the end of the experiment. Birds had ad libitum access to water and feed throughout the study. The initial room temperature was maintained at 33°C upon chick placement, and after 72 h, it was decreased by 0.5°C per d until reaching an average of 24°C by d 21. Experimental diets were formulated to be isoenergetic and isonitrogenous across treatments. Diets were provided in three phases: starter (d 1 to 10), grower (d 11 to 24), and finisher (d 25 to 42), based on the nutrient requirements recommended by the Ross 308 broiler management guide (Aviagen, 2022). The feed ingredients and the calculated and analyzed nutrient values are presented in Table 2.

**Table 2:** Composition of the basal diet in the starter (d 0 to 10), grower (d 11 to 24), and finisher (d 25 to 42) phases with different fat sources [soybean oil (SO) and beef tallow (BT)].

	Starter		Grower		Finisher	
Ingredient (%)	SO	BT	SO	BT	SO	BT
Corn	53.67	53.67	57.71	57.71	62.77	62.77
Soybean meal	31.92	31.92	26.30	26.30	20.34	20.34
Corn gluten meal	5.68	5.68	6.79	6.79	7.10	7.10
Starch	-	1.10	-	1.34	-	1.57
Soybean oil	2.80	-	3.40	-	4.00	-
Beef tallow	-	2.80	-	3.40	-	4.00
Dicalcium phosphate	1.96	1.96	1.75	1.75	1.57	1.57
Calcium carbonate	1.15	1.15	1.08	1.08	1.01	1.01
L-Lysine HCl	0.43	0.43	0.42	0.42	0.44	0.44
DL-Methionine	0.26	0.26	0.20	0.20	0.18	0.18
L-Threonine	0.21	0.21	0.17	0.17	0.15	0.15
Salt	0.27	0.27	0.19	0.19	0.14	0.14
Sodium bicarbonate	0.05	0.05	0.15	0.15	0.23	0.23
Vitamin premix^1^	0.25	0.25	0.25	0.25	0.25	0.25
Mineral premix^2^	0.25	0.25	0.25	0.25	0.25	0.25
Filler (building sand)	1.10	-	1.34	-	1.57	-
Total	100	100	100	100	100	100
Calculated analysis						
Metabolizable energy (kcal/kg)	3,000	3,000	3,100	3,100	3,200	3,200
Crude protein (%)	23.00	23.00	21.50	21.50	19.50	19.50
Crude fat (%)	5.19	5.25	5.90	5.97	6.65	6.73
Digestible lysine (%)	1.28	1.28	1.15	1.15	1.03	1.03
Digestible methionine + cystine (%)	0.95	0.95	0.87	0.87	0.80	0.80
Digestible threonine (%)	0.86	0.86	0.77	0.77	0.69	0.69
Calcium (%)	0.96	0.96	0.87	0.87	0.79	0.79
Available phosphorus (%)	0.48	0.48	0.43	0.43	0.40	0.40
DEB (mEq/kg)	250	250	235	235	220	220
Analyzed value^3^						
Gross energy (kcal/kg)	4,308 ± 23	4,295 ± 20	4,450 ± 25	4,423 ± 19	4,568 ± 17	4,537 ± 24
Crude protein (%)	22.5 ± 0.4	22.6 ± 0.3	21.2 ± 0.4	21.1 ± 0.6	19.1 ± 0.3	19.2 ± 0.3
Crude fat (%)	4.8 ± 0.1	4.7 ± 0.1	5.9 ± 0.1	5.7 ± 0.1	6.9 ± 0.1	6.7 ± 0.1
Fatty acid profile (% of total fatty acids)						
16:0	10.8 ± 0.3	19.3 ± 0.2	10.7 ± 0.3	21.1 ± 0.4	10.5 ± 0.2	22.0 ± 0.3
18:0	3.1 ± 0.1	5.7 ± 0.1	3.3 ± 0.1	6.0 ± 0.1	3.4 ± 0.1	6.3 ± 0.2
18:1n-9	22.5 ± 0.3	29.2 ± 0.2	22.5 ± 0.3	32.9 ± 0.4	22.5 ± 0.2	32.1 ± 0.4
18:2n-6	56.9 ± 0.5	40.8 ± 0.3	56.5 ± 0.6	37.2 ± 0.3	56.2 ± 0.5	34.6 ± 0.2
18:3n-3	4.41 ± 0.14	1.72 ± 0.04	4.80 ± 0.10	1.52 ± 0.04	5.18 ± 0.12	1.37 ± 0.05

1 Vitamin premix content per kg diet: 9000 IU vitamin A, 3000 IU vitamin D₃, 18 IU vitamin E, 3 mg vitamin K₃, 1.8 mg vitamin B₁ (thiamine), 6 mg vitamin B₂ (riboflavin), 3 mg vitamin B₆ (pyridoxine), 0.012 mg vitamin B₁₂ (cyanocobalamin), 30 mg vitamin B₃ (niacin), 1 mg vitamin B₉ (folic acid), 0.24 mg vitamin H₂ (biotin), 10 mg vitamin B₅ (pantothenic acid), and 500 mg choline.

2 Mineral premix content per kg diet: 100 mg manganese, 100 mg zinc, 80 mg iron, 10 mg copper, 1 mg iodine, and 0.2 mg selenium.

3 Mean ± standard deviations of three samples per diet.

### Growth performance records

Each pen (n = 5 pens/treatment; 20 birds/pen) was the experimental unit for performance. Body weight (BW) by pen and feed intake (FI) by pen were recorded on d 1 and 42 to compute body weight gain (BWG) and gain-to-feed ratio (G:F = BWG/FI). Mortality was checked at least twice daily. Performance variables were corrected for mortality by accounting for both BW at removal of dead birds in pen BWG and the estimated FI of dead birds in pen FI. The European Production Index (EPI) was calculated as:

EPI=[Livability(%)×FinalBW(kg)×100]/[Age(d)×(BWG/FI)]

### Chemical analysis and fatty acid profile measurement of breast and thigh muscles

On d 42, two birds per pen were euthanized by cervical dislocation; carcasses were defeathered and eviscerated. Breast (pectoralis major) and thigh muscles (skinless) were removed, trimmed of visible connective tissue, weighed and minced; subsamples (50 g) of breast and thigh muscles were vacuum-packed and stored at −20°C for proximate analysis and fatty acid profiling.

Proximate composition followed AOAC procedures (2006): moisture (method 934.01), crude protein [Kjeldahl N (method 990.03) × 6.25], ether extract (method 920.39), and ash (method 942.05). The pH of breast and thigh meat was determined by homogenizing 5 g of raw meat with 25 mL of distilled water. The homogenate was filtered, and the pH was measured using a NWKbinar pH meter (K-21, Landsberg, Germany) at room temperature. The device was calibrated with pH 4 and pH 7 standard buffer solutions (Ingold Messtechnik AG, Udorf, Switzerland) at 2°C after every five measurements (Jang et al., 2008).

Lipids from breast and thigh muscle subsamples were extracted using the Folch method (1956) with a chloroform:methanol ratio of 2:1 (v/v). Fatty acid methyl esters (FAME) were prepared by base-catalyzed transmethylation (0.5 M KOH in methanol) followed by acidification (or, alternatively, BF₃–methanol), extracted into hexane, and injected into a GC–FID equipped with a highly polar capillary column suitable for cis/trans and PUFA separation (Ghasemi et al., 2016). Injector and detector temperatures were approximately 250–270°C; the oven program was ramped to resolve C14:0–C22:6. Peaks were identified against a certified FAME standard mix; data were expressed as a percentage of total identified fatty acids, and indices were calculated: SFA, MUFA, PUFA (total, n-6, n-3), PUFA/SFA ratio, and n-6/n-3 ratio.

#### Digestive Enzyme Activity Assay

On d 42, small-intestinal digesta were collected immediately by gentle finger-stripping of the designated intestinal segments (two birds per replicate). Each sample was diluted 1:10 (w/v) in ice-cold phosphate-buffered saline (PBS; pH 7.0), homogenized for 60 s, and then sonicated for three 1-min cycles with 30-s intervals on ice. Homogenates were centrifuged at 18,000 × g for 20 min at 4°C, and the resulting supernatants were aliquoted and stored at −80°C until analysis. Total protein concentration in the digesta supernatants was determined by the Lowry method using porcine serum albumin (fraction V) as the standard; enzyme activities were normalized to total protein. Amylase activity was assayed according to Somogyi (1960) using soluble starch as the substrate. Lipase activity was measured titrimetrically at pH 9 in the presence of bile salts and colipase (Junge et al., 1983). Total protease activity was assayed according to Lynn and Clevette-Radford (1984). Trypsin activity was quantified at pH 8.1 using N-α-p-toluenesulfonyl-L-arginine methyl ester (TAME) as the substrate; chymotrypsin was assayed analogously using N-benzoyl-L-tyrosine ethyl ester at pH 7.8 (Wirnt, 1974a,b). All digestive enzyme activities were expressed as International Units (IU) per mg of protein.

### Nutrient Digestibility

Chromic oxide was included in all diets at 0.30% during the last 5 days of the trial to enable indicator-based digestibility calculations. From 10 birds per treatment (two per pen, pooled by pen), the distal ileum (from Meckel’s diverticulum to the ileocecal junction) was dissected; ileal digesta were gently flushed with ice-cold distilled water into preweighed tubes, pooled by pen, immediately frozen (−20°C), then freeze-dried, ground (0.5 mm), and stored desiccated. For marker-based AMEn measurement, fresh excreta were collected by pen twice daily (d 38–42) from clean plastic trays under each pen, pooled across days, dried at 55–60°C to constant weight, ground, and stored. Diet, ileal digesta, and excreta were analyzed for dry matter, crude protein, crude fat, gross energy (GE; bomb calorimetry; Parr Instrument Company, Moline, IL), and chromium (acid digestion followed by UV–Vis spectrophotometry) according to Mueller (1956). The apparent ileal digestibility (AID) of nutrients in diets was calculated by the following equation:

$$\text{AID}\;\left(\%\right)=\left[1-\left(Cr_{\text{diet}}/Cr_{\text{digesta}}\right)\times \left(N_{\text{digesta}}/{\;}_{\text{diet}}\right)\right]\times 100$$

Where Cr _diet_ and N _diet_ are the concentrations of chromic oxide and nutrient in the diet (%), and Cr _digesta_ and N _digesta_ represent the concentrations of the same chromic oxide and nutrient in the ileal digesta (%).

The AMEn values of the diets were determined using the following equation (Nari and Ghasemi, 2020):

$$\text{AMEn}\;\left(\text{Kcal}/\text{kg}\;\text{of}\;\text{diet}\right)=GE_{\text{diet}}-\left[\left(GE_{\text{excreta}}\times \text{IF}\right)+8.22\times \left(N_{\text{diet}}-N_{\text{excreta}}\times \text{IF}\right)\right]$$

Where: GE _diet_ is the gross energy value in the diet (kcal/kg) and GE _excreta_ is the gross energy value in excreta (kcal/kg); IF is the indigestibility factor (Cr _diet_/Cr _excreta_); N _diet_ is nitrogen concentration in the diet (%); N _excreta_ is nitrogen concentration in excreta (%); and 8.22 is the standard correction factor for uric-acid-based N excretion in poultry.

### Intestinal Morphology

On d 42, approximately 2-cm segments were sampled from the mid-duodenum (between the pancreatic loop) and the mid-jejunum (between Meckel’s diverticulum and the ileocecal junction) from each slaughtered bird (two birds per replicate). Segments were gently flushed with saline, fixed in 10% neutral-buffered formalin (48 h), dehydrated, embedded in paraffin, and sectioned at 5 µm. Sections were stained with hematoxylin and eosin (H&E). For each segment, two nonadjacent sections per bird were analyzed; on each section, 10 well-oriented villi (intact tip, visible crypt) were measured using calibrated image-analysis software (QWinPlus, version 3.1.0; Leica Cambridge Ltd., UK): villus height (VH; tip to villus–crypt junction), crypt depth (CD; base to villus–crypt junction), villus width (VW; midpoint), and the villus height-to-crypt depth (VH/CD) ratio. Villus surface area was estimated as 2π × (VW/2) × VH (Ahmadi-Sefat et al., 2022). The mean of the 10 villi per section was treated as the bird-level value; pen means were used for analysis.

### Statistical Analysis

All data obtained from the experiment were subjected to statistical analysis using the General Linear Model (GLM) procedure of SAS software (version 9.4; SAS Institute Inc., Cary, NC, USA), according to a 2 × 2 × 2 factorial arrangement of treatments, with fixed effects of fat source (soybean oil vs. beef tallow), LPL supplementation (0 vs. 0.10%), and inulin supplementation (0 vs. 0.10%), as well as their two-way (fat source × LPL; fat source × inulin; LPL × inulin) and three-way (fat source × LPL × inulin) interactions. For performance data, the experimental unit was the pen (20 birds/pen), whereas for biochemical parameters, enzyme activities, digestibility, and tissue composition, the individual bird was considered the experimental unit. The Shapiro–Wilk test was applied to verify the normality of residuals before conducting ANOVA. When a significant interaction term was detected, Tukey’s multiple-range test was used to separate means among the corresponding treatment combinations. Statistical significance was declared at *P* ≤ 0.05, and trends were discussed at 0.05 < *P* ≤ 0.10. All results are presented as least-squares means ± pooled standard error of the mean (SEM). When a significant interaction term was detected, interpretation was based on comparisons among the corresponding treatment combinations (simple-effects interpretation), and marginal main effects for the interacting factors were not interpreted in the text.

## Results

### Growth Performance

The effects of fat source, LPL supplementation, inulin, and their interactions on growth performance indices over the 42-d experimental period are summarized in Table 3. The three-way interaction among fat source, LPL, and inulin, as well as the two-way interaction between fat source and LPL, did not have significant effects on any of the performance parameters. In contrast, a significant interaction between fat source and inulin was observed for BWG, mortality, and the EPI (*P* < 0.05). Furthermore, a significant interaction between LPL supplementation and inulin was detected for FI and G:F (*P* < 0.05). Because these performance traits showed significant interactions, interpretation was based on the interaction patterns. Inulin supplementation improved BWG and EPI and reduced mortality in soybean-oil–based diets relative to the corresponding tallow-based diets (*P* < 0.05). In addition, within LPL-containing diets, inulin increased FI and improved G:F (*P* < 0.05).

**Table 3:** Effects of fat source [soybean oil (SO) and beef tallow (BT)] and dietary supplementation with a lysophospholipid blend (LPL) and inulin on growth performance parameters^1^ of broiler chickens from 1 to 42 d of age.

Fat source	LPL (g/kg)	Inulin (g/kg)	BWG (g)	FI (g)	G:F	Mortality (%)	EPI
SO	-	-	2,355	4,701	0.501	4.00	274.7
SO	-	+	2,492	4,719	0.528	1.33	314.6
SO	+	-	2,392	4,500	0.532	2.67	300.0
SO	+	+	2,492	4,709	0.529	0.00	319.2
BT	-	-	2,389	4,705	0.508	0.00	294.2
BT	-	+	2,346	4,523	0.520	1.33	291.2
BT	+	-	2,406	4,507	0.534	0.00	311.1
BT	+	+	2,420	4,626	0.523	2.67	298.5
SEM			24.7	68.0	0.006	1.415	6.31
Fat × inulin							
SO (-inulin)			2,374^b^			3.33^a^	287.3^c^
SO (+inulin)			2,492^a^			0.67^b^	316.9^a^
BT (-inulin)			2,397^b^			0.00^b^	302.6^b^
BT (+inulin)			2,383^b^			2.00^ab^	294.9^b^
SEM			17.4			1.000	4.46
LPL × inulin							
-LPL (-inulin)				4,703^a^	0.505^b^		
-LPL (+inulin)				4,621^ab^	0.524^a^		
+LPL (-inulin)				4,503^b^	0.533^a^		
+LPL (+inulin)				4,668^a^	0.526^a^		
SEM				48.1	0.004		
Main effect means							
Fat source							
SO			2,433^a^	4,657	0.523	2.00	302.1
BT			2,390^b^	4,590	0.521	1.00	298.7
SEM			12.3	34.0	0.003	0.707	3.15
LPL (g/kg)							
-			2,396	4,662	0.514^b^	1.67	293.6^b^
+			2,427	4,585	0.530^a^	1.33	307.2^a^
SEM			12.3	34.0	0.003	0.707	3.15
Inulin (g/kg)							
-			2,386^b^	4,603	0.519	1.67	295.0^b^
+			2,437^a^	4,644	0.525	1.33	305.9^a^
SEM			12.3	34.0	0.003	0.707	3.15
Significance							
Fat source			0.020	0.172	0.796	0.325	0.459
LPL			0.080	0.121	0.001	0.741	0.005
inulin			0.006	0.399	0.159	0.741	0.020
Fat × LPL			0.450	0.548	0.907	0.325	0.749
Fat × inulin			0.001	0.141	0.165	0.026	<0.001
LPL × inulin			0.796	0.015	0.005	0.741	0.099
Fat × LPL × inulin			0.183	0.569	0.673	0.741	0.543

a,b Means within each column with no common superscript differ (*P* < 0.05).

1 BWG, Body weight gain; FI, feed intake, G:F, gain-to-feed ratio; EPI, European production index.

### Chemical Composition and pH of Breast and Thigh Muscles

The chemical composition of breast and thigh muscles is presented in Table 4. No significant three-way interaction among fat source, LPL, and inulin, or two-way interactions between fat source and inulin or between LPL and inulin, were observed for either muscle type. However, a significant fat source × LPL interaction was detected for breast muscle fat content, such that LPL supplementation in tallow-based diets reduced breast fat content (*P* < 0.05), whereas no comparable reduction was evident in soybean-oil–based diets. For traits without significant interaction terms, replacing soybean oil with beef tallow increased thigh crude fat content and increased breast pH while reducing breast protein content (*P* < 0.05). LPL supplementation increased protein content in both breast and thigh muscles (*P* < 0.05), whereas its effect on breast fat deposition depended on fat source as described above. Additionally, inulin supplementation reduced fat content in both muscle types and significantly increased thigh muscle ash content (*P* < 0.05).

**Table 4:** Effects of fat source [soybean oil (SO) and beef tallow (BT)] and dietary supplementation with a lysophospholipid blend (LPL) and inulin on chemical composition^1^ and pH of breast and thigh muscles in broiler chickens at 42 d of age.

Fat source	LPL (g/kg)	Inulin (g/kg)	Thigh (%)					Breast (%)				
			DM	CF	CP	Ash	pH	DM	CF	CP	Ash	pH
SO	-	-	30.45	10.92	17.79	1.51	6.22	26.67	3.30	20.98	1.22	5.87
SO	-	+	30.98	10.86	18.20	1.67	6.09	27.01	3.09	21.42	1.31	5.84
SO	+	-	30.93	10.61	18.53	1.57	6.34	26.94	3.15	21.39	1.28	5.75
SO	+	+	30.91	10.47	18.56	1.64	6.15	27.30	3.17	21.63	1.33	5.75
BT	-	-	31.25	12.01	17.44	1.59	6.48	27.02	4.03	20.61	1.26	6.21
BT	-	+	31.01	11.10	18.02	1.64	6.23	26.77	3.54	20.84	1.22	5.98
BT	+	-	31.35	11.22	18.27	1.63	6.27	27.12	3.39	21.28	1.30	6.07
BT	+	+	31.12	10.84	18.38	1.66	6.27	27.26	3.48	21.23	1.37	5.94
SEM			0.359	0.234	0.327	0.050	0.143	0.258	0.077	0.251	0.067	0.088
Fat × LPL												
SO (-LPL)									3.20^c^			
SO (+LPL)									3.16^c^			
BT (-LPL)									3.79^a^			
BT (+LPL)									3.43^b^			
SEM									0.055			
Main effect means												
Fat source												
SO			30.82	10.72^b^	18.27	1.60	6.20	26.98	3.18^b^	21.36^a^	1.29	5.80^b^
BT			31.18	11.29^a^	18.02	1.63	6.31	27.04	3.61^a^	20.99^b^	1.29	6.05^a^
SEM			0.179	0.117	0.164	0.025	0.071	0.129	0.039	0.125	0.034	0.044
LPL (g/kg)												
-			30.92	11.22^a^	17.86^b^	1.60	6.26	26.87	3.49^a^	20.96^b^	1.25	5.97
+			31.08	10.78^b^	18.44^a^	1.62	6.26	27.15	3.29^b^	21.39^a^	1.32	5.88
SEM			0.179	0.117	0.164	0.025	0.071	0.129	0.039	0.125	0.034	0.044
Inulin (g/kg)												
-			30.99	11.19^a^	18.01	1.58^b^	6.33	26.94	3.47^a^	21.06	1.27	5.98
+			31.00	10.82^b^	18.29	1.65^a^	6.18	27.08	3.32^b^	21.28	1.31	5.88
SEM			0.179	0.117	0.164	0.025	0.071	0.129	0.039	0.125	0.034	0.044
Significance												
Fat source			0.158	0.001	0.291	0.333	0.276	0.714	<0.001	0.049	1.000	<0.001
LPL			0.548	0.012	0.019	0.541	0.984	0.126	0.001	0.023	0.156	0.143
inulin			0.966	0.032	0.235	0.036	0.170	0.425	0.010	0.228	0.406	0.120
Fat × LPL			0.845	0.598	0.937	0.780	0.390	0.963	0.007	0.547	0.519	0.911
Fat × inulin			0.345	0.111	0.782	0.293	0.860	0.275	0.344	0.482	0.617	0.212
LPL × inulin			0.596	0.484	0.366	0.421	0.624	0.585	0.100	0.503	0.754	0.620
Fat × LPL × inulin			0.585	0.368	0.910	0.676	0.457	0.623	0.127	0.911	0.418	0.761

a-c Means within each column with no common superscript differ (*P* < 0.05).

1 DM, dry matter; CF, crude fat, CP, crude protein

### Fatty acid Profile of Breast and Thigh Muscles

The fatty acid compositions of breast and thigh muscles are presented in Table 5, Table 6, respectively, with detailed profiles in Supplementary Table S1 (breast) and Supplementary Table S2 (thigh). No significant three-way or two-way interactions among the experimental factors (fat source, LPL, and inulin) were observed for the fatty acid profile of breast muscle. In contrast, a significant interaction between fat source and LPL was identified for n-3 PUFA concentration and the n-6/n-3 PUFA ratio in thigh muscle (Table 6). Specifically, LPL supplementation in tallow-based diets significantly increased n-3 PUFA concentration and decreased the n-6/n-3 PUFA ratio (*P* < 0.05), whereas no significant effect was observed in soybean-oil–based diets. Because thigh n-3 PUFA and the n-6/n-3 ratio were governed by a fat source × LPL interaction, these outcomes are interpreted only by treatment combinations as described above. For breast muscle (no significant interactions), replacing soybean oil with beef tallow increased SFA and MUFA and decreased n-6 PUFA, total PUFA, and the PUFA/SFA ratio (*P* < 0.05), while LPL decreased MUFA and increased n-6 PUFA and n-3 PUFA as well as the PUFA/SFA ratio (*P* < 0.05). Inulin supplementation significantly decreased SFA concentration and increased the PUFA/SFA ratio in both breast and thigh muscles (*P* < 0.05).

**Table 5:** Effects of fat source [soybean oil (SO) and beef tallow (BT)] and dietary supplementation with lysophospholipid blend (LPL) and inulin on breast fatty acid profile^1^ (% of total fatty acids) in broiler chickens at 42 d of age.

Fat source	LPL (g/kg)	Inulin (g/kg)	SFA	MUFA	n-6 PUFA	n-3 PUFA	n-6/n-3 PUFA	Total PUFA	PUFA/SFA
SO	-	-	27.40	35.46	35.32	1.81	20.68	37.14	1.35
SO	-	+	26.81	34.84	36.43	1.93	19.68	38.35	1.43
SO	+	-	27.03	32.93	38.00	2.04	18.68	40.04	1.48
SO	+	+	25.52	34.44	37.85	2.19	17.87	40.04	1.57
BT	-	-	31.88	39.27	27.47	1.38	20.10	28.85	0.91
BT	-	+	30.56	38.98	29.14	1.32	22.32	30.46	1.00
BT	+	-	31.76	36.97	29.58	1.68	18.09	31.27	0.99
BT	+	+	30.04	37.40	30.96	1.61	19.72	32.57	1.09
SEM			0.424	1.115	1.149	0.136	1.807	1.117	0.044
Main effect means									
Fat source									
SO			26.69^b^	34.42^b^	36.90^a^	1.99^a^	19.23	38.89^a^	1.46^a^
BT			31.06^a^	38.16^a^	29.29^b^	1.50^b^	20.06	30.78^b^	0.99^b^
SEM			0.212	0.557	0.574	0.068	0.904	0.559	0.022
LPL (g/kg)									
-			29.16	37.14^a^	32.09^b^	1.61^b^	20.70	33.70^b^	1.17^b^
+			28.59	35.44^b^	34.10^a^	1.88^a^	18.59	35.98^a^	1.28^a^
SEM			0.212	0.557	0.574	0.068	0.904	0.559	0.022
Inulin (g/kg)									
-			29.52^a^	36.16	32.59	1.73	19.39	34.32	1.18^b^
+			28.23^b^	36.42	33.59	1.76	19.90	35.35	1.27^a^
SEM			0.212	0.557	0.574	0.068	0.904	0.559	0.022
Significance									
Fat source			<0.001	<0.001	<0.001	<0.001	0.521	<0.001	<0.001
LPL			0.064	0.038	0.019	0.009	0.109	0.007	0.001
inulin			<0.001	0.745	0.227	0.750	0.691	0.202	0.008
Fat × LPL			0.401	0.764	0.960	0.797	0.875	0.983	0.440
Fat × inulin			0.442	0.816	0.525	0.307	0.276	0.597	0.837
LPL × inulin			0.282	0.376	0.638	0.959	0.941	0.633	0.887
Fat × LPL × inulin			0.674	0.656	0.770	0.902	0.880	0.776	0.962

a,b Means within each column with no common superscript differ (*P* < 0.05). The detailed breast fatty acid profile is presented in a Supplementary Table S1.

1 SFA, saturated fatty acids (C14:0 + C16:0 + C18:0); MUFA, monounsaturated fatty acids (C16:1 + C18:1 + C20:1); n-6 PUFA, n-6 polyunsaturated fatty acids (C18:2 + C20:4); n-3 PUFA, n-3 polyunsaturated fatty acids (C18:3 + C20:5 + C22:6); Total PUFA, total polyunsaturated fatty acids (n-6 PUFA + n-3 PUFA).

**Table 6:** Effects of fat source [soybean oil (SO) and beef tallow (BT)] and dietary supplementation with a lysophospholipid blend (LPL) and inulin on thigh fatty acid profile^1^ (% of total fatty acids) in broiler chickens at 42 d of age.

Fat source	LPL (g/kg)	Inulin (g/kg)	SFA	MUFA	n-6 PUFA	n-3 PUFA	n-6/n-3 PUFA	Total PUFA	PUFA/SFA
SO	-	-	25.08	37.32	35.69	1.91	19.56	37.61	1.50
SO	-	+	24.25	36.91	36.81	2.02	18.64	38.83	1.60
SO	+	-	25.06	35.77	37.01	2.16	17.61	39.17	1.57
SO	+	+	23.56	36.43	37.90	2.11	18.31	40.01	1.70
BT	-	-	30.67	39.58	28.64	1.12	26.18	29.75	0.97
BT	-	+	28.11	38.90	31.91	1.08	30.35	32.99	1.18
BT	+	-	30.29	37.06	30.89	1.75	18.18	32.65	1.08
BT	+	+	27.75	37.47	33.09	1.69	20.52	34.78	1.26
SEM			0.507	1.310	1.386	0.140	2.228	1.326	0.061
Fat × LPL									
SO (-LPL)						1.97^ab^	19.10^b^		
SO (+LPL)						2.13^a^	17.96^b^		
BT (-LPL)						1.10^c^	28.27^a^		
BT (+LPL)						1.72^b^	19.35^b^		
SEM						0.099	1.576		
Main effect means									
Fat source									
SO			24.49^b^	36.61	36.85^a^	2.05^a^	18.53^b^	38.90^a^	1.59^a^
BT			29.21^a^	38.25	31.13^b^	1.41^b^	23.81^a^	32.54^b^	1.12^b^
SEM			0.253	0.655	0.693	0.070	1.114	0.663	0.031
LPL (g/kg)									
-			27.03	38.18	33.26	1.53^b^	23.68^a^	34.80	1.31^b^
+			26.67	36.68	34.72	1.93^a^	18.66^b^	36.65	1.40^a^
SEM			0.253	0.655	0.693	0.070	1.114	0.663	0.031
Inulin (g/kg)									
-			27.77^a^	37.43	33.06	1.74	20.38	34.79	1.28^b^
+			25.92^b^	37.43	34.93	1.73	21.96	36.65	1.43^a^
SEM			0.253	0.655	0.693	0.070	1.114	0.663	0.031
Significance									
Fat source			<0.001	0.086	<0.001	<0.001	0.002	<0.001	<0.001
LPL			0.323	0.117	0.145	<0.001	0.003	0.056	0.050
inulin			<0.001	0.997	0.066	0.924	0.326	0.056	0.001
Fat × LPL			0.986	0.609	0.795	0.027	0.019	0.610	0.882
Fat × inulin			0.061	0.887	0.384	0.708	0.294	0.384	0.420
LPL × inulin			0.651	0.565	0.742	0.643	0.973	0.693	0.991
Fat × LPL × inulin			0.628	0.997	0.829	0.738	0.588	0.851	0.758

a-c Means within each column with no common superscript differ (*P* < 0.05). The detailed thigh fatty acid profile is presented in a Supplementary Table S2.

1 SFA, saturated fatty acids (C14:0 + C16:0 + C18:0); MUFA, monounsaturated fatty acids (C16:1 + C18:1 + C20:1); n-6 PUFA, n-6 polyunsaturated fatty acids (C18:2 + C20:4); n-3 PUFA, n-3 polyunsaturated fatty acids (C18:3 + C20:5 + C22:6); Total PUFA, total polyunsaturated fatty acids (n-6 PUFA + n-3 PUFA).

### Digestive Enzyme Activity

The activities of digestive enzymes in the small intestine are presented in Table 7. No significant main effect of fat source was detected, nor were any significant two- or three-way interactions among the experimental factors (fat source, LPL, and inulin) observed for the activities of protease, trypsin, chymotrypsin, lipase, or amylase. In contrast, LPL supplementation significantly increased the activities of protease and lipase (*P* < 0.05). Inulin supplementation also resulted in a significant increase in protease activity (*P* = 0.033) and showed a trend toward increased amylase activity (*P* = 0.097).

**Table 7:** Effects of fat source [soybean oil (SO) and beef tallow (BT)] and dietary supplementation with a lysophospholipid blend (LPL) and inulin on intestinal digestive enzyme activities (IU/mg of digesta protein) in broiler chickens at 42 d of age.

Fat source	LPL (g/kg)	Inulin (g/kg)	Protease	Trypsin	Chymotrypsin	Lipase	Amylase
SO	-	-	72.82	40.29	9.46	23.43	51.46
SO	-	+	79.41	47.11	10.06	24.17	59.47
SO	+	-	79.38	44.19	9.36	28.68	57.29
SO	+	+	89.02	47.36	9.05	29.46	60.59
BT	-	-	65.47	38.87	8.79	26.29	53.07
BT	-	+	80.97	43.69	9.66	26.25	60.24
BT	+	-	79.44	46.82	9.26	28.00	54.23
BT	+	+	87.62	47.89	10.14	28.74	59.20
SEM			6.344	3.923	0.591	1.751	4.852
Main effect means							
Fat source							
SO			80.16	44.74	9.48	26.43	57.20
BT			78.38	44.32	9.46	27.32	56.69
SEM			3.172	1.961	0.296	0.875	2.426
LPL (g/kg)							
-			74.67^b^	42.49	9.49	25.04^b^	56.06
+			83.86^a^	46.57	9.45	28.72^a^	57.83
SEM			3.172	1.961	0.296	0.875	2.426
Inulin (g/kg)							
-			74.28^b^	42.54	9.22	26.60	54.01
+			84.25^a^	46.51	9.73	27.16	59.87
SEM			3.172	1.961	0.296	0.875	2.426
Significance							
Fat source			0.694	0.880	0.962	0.477	0.882
LPL			0.049	0.151	0.919	0.006	0.610
inulin			0.033	0.162	0.232	0.656	0.097
Fat × LPL			0.806	0.476	0.226	0.209	0.622
Fat × inulin			0.681	0.714	0.393	0.869	0.953
LPL × inulin			0.813	0.508	0.591	0.868	0.619
Fat × LPL × inulin			0.567	0.993	0.583	0.883	0.857

a,b Means within each column with no common superscript differ (*P* < 0.05).

### Nutrient Digestibility

The effects of fat source, LPL supplementation, inulin, and their interactions on nutrient digestibility and AMEn are presented in Table 8. No significant three-way interaction among fat source, LPL, and inulin, nor any significant two-way interactions between fat source and inulin or between LPL and inulin, were observed for nutrient digestibility or AMEn. In contrast, a significant interaction between fat source and LPL was detected for fat digestibility and AMEn (*P* < 0.05). Accordingly, interpretation focused on the fat source × LPL interaction: LPL increased fat digestibility and AMEn in tallow-based diets, whereas corresponding changes were minimal in soybean-oil–based diets (*P* < 0.05). Inulin supplementation significantly improved protein digestibility (*P* < 0.05) and showed a trend toward improved ash digestibility (*P* = 0.098).

**Table 8:** Effects of fat source [soybean oil (SO) and beef tallow (BT)] and dietary supplementation with a lysophospholipid blend (LPL) and inulin on nutrient digestibility^1^ in broiler chickens at 42 d of age.

			DM	CP	CF	Ash	Energy	AMEn
Fat source	LPL (g/kg)	Inulin (g/kg)	%	%	%	%	%	Kcal/kg
SO	-	-	75.91	68.70	82.54	53.02	74.46	3165
SO	-	+	77.07	70.78	82.78	54.59	74.35	3186
SO	+	-	77.42	72.12	83.04	52.99	74.92	3208
SO	+	+	77.46	72.70	83.42	54.56	75.08	3206
BT	-	-	73.96	68.92	77.98	51.35	70.82	3077
BT	-	+	75.36	70.07	78.44	52.89	71.31	3086
BT	+	-	75.32	70.21	81.84	53.96	73.69	3182
BT	+	+	76.35	71.66	82.34	54.65	74.17	3195
SEM			0.987	0.819	1.008	1.114	0.868	20.3
Fat × LPL								
SO (-LPL)					82.66^a^			3176^a^
SO (+LPL)					83.23^a^			3207^a^
BT (-LPL)					78.21^b^			3082^b^
BT (+LPL)					82.09^a^			3189^a^
SEM					0.713			14.4
Main effect means								
Fat source								
SO			76.96^a^	71.07	82.94^a^	53.79	74.70^a^	3191^a^
BT			75.25^b^	70.22	80.15^b^	53.21	72.50^b^	3135^b^
SEM			0.493	0.410	0.504	0.557	0.434	10.2
LPL (g/kg)								
-			75.58	69.62^b^	80.44^b^	52.96	72.73^b^	3129^b^
+			76.64	71.67^a^	82.66^a^	54.04	74.47^a^	3198^a^
SEM			0.493	0.410	0.504	0.557	0.434	10.2
Inulin (g/kg)								
-			75.65	69.99^b^	81.35	52.83	73.47	3158
+			76.56	71.30^a^	81.75	54.17	73.73	3168
SEM			0.493	0.410	0.504	0.557	0.434	10.2
Significance								
Fat source			0.020	0.148	<0.001	0.467	0.001	0.001
LPL			0.138	0.001	0.004	0.180	0.008	<0.001
inulin			0.202	0.030	0.581	0.098	0.679	0.483
Fat × LPL			0.873	0.296	0.027	0.170	0.074	0.013
Fat × inulin			0.664	0.984	0.908	0.776	0.710	0.951
LPL × inulin			0.594	0.609	0.949	0.789	0.918	0.746
Fat × LPL × inulin			0.789	0.441	0.971	0.787	0.907	0.640

a,b Means within each column with no common superscript differ (*P* < 0.05).

1 DM, dry matter; CF, crude fat, CP, crude protein; AMEn, apparent metabolizable energy corrected for nitrogen retention.

### Intestinal Morphology

The results related to intestinal histomorphology in the duodenum and jejunum are presented in Table 9. No significant main effect of fat source was detected, nor were any significant three-way (fat source × LPL × inulin) or two-way (fat source × LPL; fat source × inulin; LPL × inulin) interactions observed for any of the evaluated morphological parameters. In contrast, LPL supplementation significantly increased VH and VSA in the duodenum (*P* < 0.05). Additionally, inulin supplementation significantly improved duodenal VSA and increased VH, the VH/CD ratio, and VSA in the jejunum (*P* < 0.05).

**Table 9:** Effects of fat source [soybean oil (SO) and beef tallow (BT)] and dietary supplementation with a lysophospholipid blend (LPL) and inulin on intestinal morphology^1^ in broiler chickens at 42 d of age.

Fat source	LPL (g/kg)	Inulin (g/kg)	Duodenum					Jejunum				
			VH	CD	VW		VSA	VH	CD	VW		VSA
			µm	µm	µm	VH/CD	mm^2^	µm	µm	µm	VH/CD	mm^2^
SO	-	-	1588	168.5	122.2	9.98	0.609	1070	147.3	115.3	7.33	0.387
SO	-	+	1753	157.4	123.1	11.76	0.678	1201	143.1	123.6	8.76	0.455
SO	+	-	1794	160.9	125.4	11.34	0.703	1084	146.0	124.3	7.56	0.425
SO	+	+	1779	152.9	126.5	11.72	0.707	1173	125.0	134.9	9.68	0.502
BT	-	-	1551	167.4	111.2	9.31	0.544	1049	137.9	119.1	7.68	0.392
BT	-	+	1683	165.8	128.5	10.60	0.680	1215	133.3	123.3	9.39	0.472
BT	+	-	1786	159.7	124.0	11.22	0.690	1158	134.2	117.8	8.70	0.434
BT	+	+	1807	154.5	133.4	11.74	0.752	1199	131.9	129.5	9.25	0.484
SEM			75.7	12.61	7.10	0.957	0.0434	65.6	9.33	8.94	0.809	0.0401
Main effect means												
Fat source												
SO			1729	159.9	124.3	11.20	0.674	1132	140.4	124.5	8.33	0.442
BT			1707	161.9	124.3	10.72	0.667	1155	134.3	122.4	8.76	0.445
SEM			37.8	6.30	3.55	0.479	0.0217	32.8	4.66	4.47	0.405	0.0200
LPL (g/kg)												
-			1644^b^	164.8	121.2	10.41	0.628^b^	1134	140.4	120.3	8.29	0.426
+			1791^a^	157.0	127.3	11.51	0.713^a^	1154	134.3	126.7	8.80	0.461
SEM			37.8	6.30	3.55	0.479	0.0217	32.8	4.66	4.47	0.405	0.0200
Inulin (g/kg)												
-			1680	164.1	120.7	10.46	0.636^b^	1090^b^	141.4	119.1	7.82^b^	0.410^b^
+			1756	157.6	127.9	11.45	0.704^a^	1197^a^	133.3	127.8	9.27^a^	0.478^a^
SEM			37.8	6.30	3.55	0.479	0.0217	32.8	4.66	4.47	0.405	0.0200
Significance												
Fat source			0.685	0.827	0.993	0.479	0.811	0.618	0.368	0.743	0.465	0.911
LPL			0.010	0.391	0.234	0.116	0.009	0.670	0.360	0.324	0.379	0.227
inulin			0.165	0.472	0.162	0.153	0.034	0.028	0.231	0.178	0.016	0.021
Fat × LPL			0.557	0.846	0.594	0.528	0.447	0.569	0.594	0.547	0.909	0.790
Fat × inulin			0.988	0.732	0.226	0.896	0.315	0.947	0.492	0.908	0.575	0.897
LPL × inulin			0.185	0.987	0.698	0.430	0.266	0.371	0.583	0.701	0.842	0.856
Fat × LPL × inulin			0.753	0.854	0.688	0.819	0.946	0.661	0.476	0.843	0.426	0.719

a,b Means within each column with no common superscript differ (*P* < 0.05).

1 VH, villus height, CD, crypt depth; VW, villus width; VH/CD, villus height/crypt depth ratio; VSA, villus surface area.

## Discussion

The present study demonstrated that both the type of dietary fat and the inclusion of functional additives, such as inulin and emulsifiers, exerted measurable effects on broiler growth performance during the 42-d rearing period. In the present study, growth responses were governed by significant interactions among dietary factors. The fat source × inulin interaction indicates that the effects of inulin on BWG, EPI, and mortality depended on the lipid source, with the most pronounced improvements observed when inulin was added to soybean-oil–based diets. In addition, the LPL × inulin interaction for FI and G:F suggests complementary mechanisms, whereby improved emulsification and nutrient access in the presence of LPL may enhance the performance response to inulin-mediated modulation of gut function. Accordingly, interpretation is focused on interaction patterns rather than marginal main effects for the interacting factors. Because inulin acts primarily via hindgut fermentation, its benefits are mediated through microbiota-derived SCFA that support intestinal integrity and absorptive function (Shang et al., 2018; Kareem et al., 2016), which have been linked to improved growth and survivability in broilers (Wu et al., 2019; Xia et al., 2021). These benefits may be expressed more clearly when dietary lipid is readily emulsified and absorbed (soybean oil), such that energy supply is less limiting and gut function improvements translate into measurable gains in BWG and EPI. In contrast, its benefits appeared attenuated in tallow-based diets, potentially because saturated-fat matrices can constrain emulsification and modify lipid flow to distal gut segments, which may blunt the net performance response to inulin despite its prebiotic action.

Additionally, the interaction between LPL supplementation and inulin support the hypothesis that LPL supplementation enhances micelle formation and fat utilization, whereas inulin modulates intestinal health, but the direction of the response differed by trait: inulin increased FI primarily when LPL was present, whereas improvements in G:F were more evident when LPL was not included. Because this interaction pattern was not accompanied by an increase in AMEn, the FI response is unlikely to reflect greater dietary energy provision; rather, it may indicate that inulin-mediated improvements in intestinal environment (e.g., microbiota modulation and SCFA production) supported appetite and feeding behavior when lipid digestion was facilitated by LPL. Conversely, in the absence of LPL, inulin-related improvements in nutrient extraction (e.g., improved protein digestibility and jejunal villus development observed in the current study) could explain the higher G:F without a concurrent increase in FI. Emulsifiers act by reducing surface tension between water and lipids, promoting the formation of smaller lipid droplets and more stable micelles, thereby improving the emulsification and digestion of dietary fats and fat-soluble nutrients (Haetinger et al., 2021; Wang et al., 2024). In the current study, these mechanisms are consistent with the observed improvement in FI, EPI, and the tendency toward increased BWG when emulsifiers were added. Similar positive responses in BWG and feed efficiency to LPL supplementation in broilers fed low-energy diets have been reported (Ghazanfari et al., 2024; Pashaei Jalal et al., 2024), strengthening the evidence that emulsifiers are effective enhancers of energy utilization.

The chemical composition and pH of breast and thigh muscles were modulated by dietary fat source and functional additives, with no evidence of a three-way interaction and no significant fat source × inulin or LPL × inulin interactions, indicating that additive effects for most endpoints were consistent across the other factors. Notably, breast fat content was governed by a fat source × LPL interaction, such that LPL supplementation reduced breast fat only in tallow-based diets. This response is biologically plausible because lysophospholipids are amphiphilic molecules that lower the oil–water interfacial tension, promote formation of smaller lipid droplets and mixed micelles, and facilitate pancreatic lipase action—effects that are expected to be most beneficial when saturated, higher–melting point fats (e.g., tallow) limit emulsification and absorption. Accordingly, improved lipid utilization under tallow feeding may alter nutrient partitioning toward lean deposition and reduce the relative accumulation of intramuscular lipid, which is consistent with the tallow-specific reduction in breast fat observed here and with prior emulsifier studies (Shoaib et al., 2022; Ye et al., 2022). For variables without significant interaction terms, the substitution of soybean oil with tallow increased crude fat in thigh muscle and increased both fat content and pH in breast muscle, while concurrently reducing protein content. Importantly, greater intramuscular fat concentration under tallow does not necessarily conflict with modestly lower lipid-utilization efficiency, because muscle ether extract reflects both lipid deposition and the extent of lean (protein) accretion; saturated-fat feeding can promote lipid retention while depressing protein deposition, thereby increasing ether extract concentration on a percentage basis. These findings align with reports that diets high in saturated fats can elevate intramuscular lipid deposition while depressing protein accretion (Sanz, 1999). The elevated breast pH observed with tallow may reflect altered muscle glycogen availability and postmortem glycolysis, as described in mechanistic frameworks linking substrate status to ultimate pH (Matarneh et al., 2018). Across diets, LPL supplementation increased protein content in both breast and thigh muscles, suggesting improved nutrient absorption and lean-tissue accretion, while its influence on breast fat depended on the accompanying fat source (Liu et al., 2023). Similarly, inulin supplementation reduced fat content in both muscle types and increased thigh ash content, which is biologically plausible because inulin is fermented to SCFA that can influence host lipid metabolism (e.g., reduced hepatic lipogenesis and altered nutrient partitioning) and improve intestinal integrity (Yan et al., 2025), thereby reducing conditions that favor fat deposition. In addition, fermentation-associated reductions in digesta pH and microbiota-mediated changes can enhance mineral solubility and absorption, providing a plausible explanation for the increased thigh ash content (Obianwuna et al., 2023). Collectively, these findings indicate that interpretation of breast fat deposition should be interaction-based (fat source × LPL), whereas other composition and pH responses may be discussed as overall effects because interaction terms were not significant.

The fatty acid composition of broiler meat is a major determinant of its nutritional value, and it can be markedly altered by dietary lipid source and functional additives. In the current study, fat source was the primary driver of breast and thigh fatty acid profiles, with tallow increasing SFA and MUFA and decreasing total PUFA (including n-6 and n-3), thereby lowering the PUFA/SFA ratio compared with soybean oil; this pattern is consistent with the lower degree of unsaturation and reduced emulsifiability of animal fats, which can constrain intestinal lipid digestion and subsequent PUFA deposition in tissues (Kanakri et al., 2018; Vakili and Ebrahimnezhad, 2023). Conversely, soybean oil promoted PUFA enrichment in muscle, reflecting its higher linoleic and linolenic acid content and the efficient transfer of unsaturated fatty acids from diet to edible tissues (Saleh et al., 2021). Importantly, interpretation of LPL effects differs by muscle and response variable: for breast fatty acids, no interaction terms were detected, supporting discussion of overall LPL-associated shifts (e.g., reduced MUFA with increased PUFA; Table 5), whereas in thigh muscle a significant fat source × LPL interaction governed n-3 PUFA concentration and the n-6/n-3 ratio, requiring an interaction-based interpretation rather than marginal main effects. Specifically, LPL supplementation increased thigh n-3 PUFA and decreased the n-6/n-3 ratio in tallow-based diets, while similar changes were not observed in soybean-oil–based diets, indicating that the efficacy of lysophospholipids depends on the accompanying lipid matrix (Ye et al., 2022). Mechanistically, lysophospholipids can enhance emulsification, micelle formation, and intestinal lipid transport, which is expected to be most beneficial when lipid emulsification is limiting—such as in saturated, higher–melting point fats—thereby improving fatty acid absorption and facilitating deposition of nutritionally favorable PUFA fractions under tallow feeding (Ye et al., 2022). The absence of comparable responses in soybean-oil diets likely reflects the inherently high digestibility and emulsifiability of unsaturated oils, which reduces the incremental value of exogenous emulsification support. Inulin supplementation also improved meat lipid quality by decreasing SFA and increasing the PUFA/SFA ratio in both breast and thigh muscles, consistent with reports that prebiotic fibers can influence lipid metabolism through microbiota-driven fermentation and SCFA production, with downstream effects on hepatic lipogenesis and tissue fatty acid accretion (Kareem et al., 2016; Grela et al., 2021). From a human nutrition perspective, the observed increases in PUFA/SFA ratio and the interaction-dependent reduction in thigh n-6/n-3 ratio—most evident when LPL was combined with tallow—are desirable, as lower n-6/n-3 ratios are commonly associated with more favorable inflammatory and cardiometabolic profiles (Heshmati, 2021). Collectively, these results underscore that LPL-related improvements in thigh n-3 PUFA and n-6/n-3 ratio should be interpreted through the fat source × LPL interaction, while inulin-associated shifts appear consistent across fat sources, supporting targeted formulation strategies to enhance both broiler performance and the nutritional quality of meat.

The present study showed that neither fat source nor its interactions with LPL or inulin supplementation significantly influenced the activity of key digestive enzymes in the small intestine, which is consistent with earlier reports indicating that dietary lipid type exerts only marginal effects on enzyme secretion when overall nutrient balance is maintained (Attia et al., 2020; Elbaz et al., 2023). Instead, dietary LPL supplementation significantly increased protease and lipase activities (Table 7), supporting the notion that emulsifiers facilitate micelle formation and substrate access, thereby stimulating pancreatic secretions and improving nutrient hydrolysis (Bai et al., 2019; Pizones Ruiz-Henestrosa et al., 2022). Although LPL is primarily intended to improve lipid emulsification, pancreatic exocrine secretion is coordinated; therefore, improved emulsification and nutrient sensing in the proximal intestine may increase secretion and/or activity of multiple enzymes, including proteases, which is consistent with the higher crude protein digestibility observed with LPL supplementation (Table 8). This observation aligns with findings that emulsifiers increase lipid digestibility and enzyme efficiency by reducing surface tension at the oil–water interface (Michels et al., 2025). Inulin supplementation further promoted protease activity and tended to increase amylase activity, which may reflect indirect gut–pancreas signaling; although inulin is primarily fermented in the ceca, fermentation-derived metabolites (e.g., SCFA) and microbiota-driven endocrine responses can influence proximal intestinal function and pancreatic exocrine secretion, thereby affecting enzyme activities measured in small-intestinal digesta rather than indicating direct fermentation in the sampled segments (Liu et al., 2018; Özlüer Hunt et al., 2019). Comparable improvements in enzymatic activity following prebiotic supplementation have been documented in broilers (Tang et al., 2025) and catfish (Zhang et al., 2023), where shifts in gut microbiota and metabolite profiles enhanced digestive efficiency. It is plausible that the enhancement of digestive capacity by LPL and inulin, through complementary physicochemical and microbiota-mediated mechanisms, is one mechanism contributing to improved nutrient utilization and growth performance in the current study.

The present study demonstrated that dietary fat source, LPL supplementation, and inulin inclusion exerted distinct effects on broiler nutrient digestibility and AMEn. A notable fat source × LPL interaction indicated that the efficacy of LPL depended on dietary lipid type. Specifically, a fat source × LPL interaction for fat digestibility and AMEn indicates that the magnitude of the LPL response depended on dietary lipid type, and therefore marginal main effects for these endpoints are not interpreted. Because soybean oil and beef tallow were supplied as added (free) fats rather than as lipids embedded within intact feed ingredients, baseline fat digestibility was expected to be relatively high, thereby limiting the magnitude of improvement achievable with emulsifier supplementation. In practical terms, tallow-based diets without LPL exhibited lower fat digestibility and AMEn than soybean-oil–based diets, consistent with the higher saturation and melting point of animal fats, which can impair emulsification and micelle formation and thereby limit intestinal lipid hydrolysis and absorption (Ye et al., 2022; Shoaib et al., 2023). LPL supplementation effectively counteracted these limitations by enhancing micelle formation and fatty acid absorption, leading to modest but statistically detectable improvements in fat digestibility and AMEn in tallow-based diets, which is consistent with the relatively small numerical differences observed in Table 8. These responses were accompanied by improvements in overall nutrient utilization, thereby supporting broader nutrient assimilation (Majdolhosseini et al., 2019; Ani et al., 2024; Mohiti-Asli et al., 2025). Accordingly, the present findings suggest that LPL benefits are most evident when lipid emulsification is a limiting step (e.g., saturated fats), whereas larger effect sizes may be expected in feeding scenarios with greater constraints on lipid digestion (e.g., younger birds, higher saturated-fat inclusion, or fats present within intact matrices. This interaction pattern is also biologically coherent with the improved performance indices observed under tallow plus LPL, as greater AMEn and lipid utilization increase energy availability for growth and can spare amino acids from oxidative metabolism. In contrast to the interaction-governed lipid outcomes, inulin supplementation improved protein digestibility and tended to increase ash digestibility, likely via its prebiotic effects on the gut microbiota and fermentation-derived SCFA, which enhance nutrient absorption and improve the gut environment (Wu et al., 2019; Obianwuna et al., 2023). Collectively, these findings underscore the importance of aligning feed additive choices with the physicochemical properties of the dietary fat source, with emulsifiers proving particularly effective in diets containing animal fats, whereas inulin primarily enhanced nitrogen retention and protein accretion.

Dietary modulation of intestinal morphology is widely recognized as a key determinant of nutrient absorption and overall performance in poultry, since VH, CD, and the VH/CD ratio collectively reflect the balance between absorptive surface area and epithelial cell turnover. Previous reports consistently indicate that improved villus height and higher VH/CD ratios are associated with enhanced absorptive capacity and growth efficiency in broilers (Khajeh Bami et al., 2022; Luo et al., 2021). {+Consistent with this framework, in the current study LPL supplementation increased duodenal villus height and mucosal surface area, whereas inulin increased jejunal villus height, the VH/CD ratio, and surface area (*P* < 0.05), indicating improved absorptive architecture in the respective intestinal segments.+} For instance, the inclusion of emulsifiers such as lysophospholipids has been shown to increase villus length in the jejunum and ileum, suggesting more efficient utilization of dietary energy and nutrients (Yousefi et al., 2023; Abbasi et al., 2025). Similarly, supplementation with prebiotic compounds has demonstrated positive modulation of gut histomorphology, often through increased villus length and mucosal surface area (Song et al., 2018; Abbasnejad Shani and Irani, 2024). However, other studies have reported limited or non-significant effects (Moreno-Mendoza et al., 2021; Teng et al., 2021), indicating that improvements in gut morphology may depend on factors such as diet composition, age, and the health status of the birds. Our findings are also consistent with previous reports (Khonyoung et al., 2015; Tenório et al., 2022), which likewise observed no notable differences in intestinal architecture when evaluating various dietary fat sources in broilers. In contrast, Azzam et al. (2025) reported that the dietary fat source could influence small intestinal morphology in a sex-dependent manner, with canola oil improving VH and the VH/CD ratio in males and rice bran oil exerting similar benefits in females. These differences could be due to several factors, including the type and level of fat, duration of fat use, basal diet, and physiological state of the birds. In this study, the absence of differences between fat sources may be attributed to the basal diet supplying adequate energy and essential nutrients, thereby minimizing the potential for additional morphological adaptations to the dietary fat source.

## Conclusions

In conclusion, both LPL and inulin exert beneficial independent effects on broiler performance, nutrient utilization, and body composition; however, their impact is strongly influenced by the dietary fat source. LPL supplementation improved nutrient digestibility and energy efficiency, particularly in tallow-based diets, whereas inulin consistently enhanced performance indices in soybean-oil–based diets. Notably, responses to LPL and inulin were dependent on dietary context, and the presence of significant interaction terms for selected performance variables indicates that the magnitude and direction of responses varied across treatment combinations. These results emphasize the importance of considering not only the additive effects of each supplement but also their synergistic interactions within specific dietary contexts. Such insights provide a practical framework for formulating more precise and cost-effective feeding strategies. Future research should explore these interactive mechanisms under commercial conditions to optimize nutrient-use efficiency and production outcomes.

## CRediT authorship contribution statement

**Mozafar Rahimpour:** Writing – original draft, Visualization, Investigation, Data curation, Conceptualization. **Kamran Taherpour:** Writing – review & editing, Writing – original draft, Supervision, Project administration, Formal analysis, Conceptualization. **Hossein Ali Ghasemi:** Writing – review & editing, Validation, Software, Methodology, Conceptualization. **Hassan Shirzadi:** Writing – review & editing, Resources, Investigation, Conceptualization.

## Disclosures

All authors approve the submission of this manuscript and declare no conflict of interest.

## Appendix Supplementary materials

Image, application 1

Image, application 2

## Footnotes

Supplementary material associated with this article can be found, in the online version, at doi:10.1016/j.psj.2026.106531.

## References

[bib0001] Abbasi M., Ghasemi H.A., Fakhraei J., Yarahmadi H.Mansoori, Hajkhodadadi I. (2025). Multifactorial evaluation of fat source, energy, and lysophospholipid supplementation on performance, metabolic profile, yolk fatty acid composition, and digestive health in peak-phase laying hens. J. Agric. Food Res..

[bib0002] Abbasnejad Shani M., Irani M. (2024). Feeding strategy and prebiotic supplementation: effects on immune responses and gut health in the early life stage of broiler chickens. Res. Vet. Sci..

[bib0003] Ahmadi-Sefat A.A., Taherpour K., Ghasemi H.A., Akbari Gharaei M., Shirzadi H., Rostami F. (2022). Effects of an emulsifier blend supplementation on growth performance, nutrient digestibility, intestinal morphology, and muscle fatty acid profile of broiler chickens fed different levels of energy and protein. Poult. Sci..

[bib0004] Albouery M., Bretin A., Buteau B., Grégoire S., Martine L., Gambert S., Bron A.M., Acar N., Chassaing B., Bringer M.-A. (2021). Soluble fiber inulin consumption limits alterations of the gut microbiota and hepatic fatty acid metabolism caused by high-fat diet. Nutrients.

[bib0005] Ani A.O., Onodugo M.O., Ogwuegbu M.C., Udeh V.C., Mthiyane D.M.N. (2024). Influence of dietary lysolecithin on growth performance, nutrient digestibility, haemato-biochemistry, and oxidative status of broiler birds. Trop. Anim. Health Prod..

[bib0006] AOAC International (2006).

[bib0007] AOCS (2011).

[bib0008] Arbabi-Motlagh M.M., Ghasemi H.A., Hajkhodadadi I., Ebrahimi M. (2022). Effect of chelated source of additional zinc and selenium on performance, yolk fatty acid composition, and oxidative stability in laying hens fed oxidised oil. Br. Poult. Sci..

[bib0009] Attia Y.A., Al-Harthi M.A., Abo El-Maaty H.M. (2020). The effects of different oil sources on performance, digestive enzymes, carcass traits, biochemical, immunological, antioxidant, and morphometric responses of broiler chicks. Front. Vet. Sci..

[bib0010] Aviagen. 2022. Ross broiler nutrition specifications 2022. https://aviagen.com/assets/Tech_Center/Ross_Broiler/Ross-BroilerNutritionSpecifications2022-[5EN.pdf.

[bib0011] Azzam M.M., Alabdullatif A.A., Alhotan R.A., Al-Badwi M.A., Akasha M.E., Dong X., Elnesr S.S., Shouman Z. (2025). Effects of palm oil, canola oil, and rice bran oil on growth performance, blood biochemistry, and intestinal morphology in young chicks. J. Appl. Poult. Res..

[bib0012] Bai G., He W., Yang Z., Fu H., Qiu S., Gao F., Shi B. (2019). Effects of different emulsifiers on growth performance, nutrient digestibility, and digestive enzyme activity in weanling pigs. J. Anim. Sci..

[bib0013] Baião N.C., Lara L.J.C. (2005). Oil and fat in broiler nutrition. Braz. J. Poult. Sci..

[bib0014] Bassareh M., Rezaeipour V., Abdullahpour R., Asadzadeh S. (2023). Dietary threonine and lysophospholipid supplement in broiler chickens: effect on productive performance, carcass variables, cecal microbiota activity, and jejunal morphology. Trop. Anim. Health Prod..

[bib0015] Buclaw M. (2017). Inulin in poultry production. World’s. Poult. Sci. J..

[bib0016] Carmona J.M., López-Bote C.J., Daza A., Rey A.I. (2017). Fat accumulation, fatty acids and melting point changes in broiler chick abdominal fat as affected by time of dietary fat feeding and slaughter age. Br. Poult. Sci..

[bib0017] Chen H., Zhou S., Li J., Huang X., Cheng J., Jiang X., Qin W., Liu Y., Liu A., Zhang Q., Lin D., Zhang Z., Chen D. (2020). Xyloglucan compounded inulin or arabinoxylan against glycometabolism disorder via different metabolic pathways: gut microbiota and bile acid receptor effects. J. Funct. Foods.

[bib0018] Dunshea F.R., Pluske J.R., Ponnampalam E.N. (2024). Dietary iron or inulin supplementation alters iron status, growth performance, intramuscular fat and meat quality in finisher pigs. Meat Sci.

[bib0019] Elbaz A.M., Zaki E.F., Salama A.A., Badri F.B., Thabet H.A. (2023). Assessing different oil sources efficacy in reducing environmental heat-stress effects via improving performance, digestive enzymes, antioxidant status, and meat quality. Sci. Rep..

[bib0020] Ferrini G., Baucells M.D., Esteve-García E., Barroeta A.C. (2008). Dietary polyunsaturated fat reduces skin fat as well as abdominal fat in broiler chickens. Poult. Sci..

[bib0021] Folch J., Lees M., Stanley G.H. (1956). A simple method for the isolation of total lipids from animal tissues. J. Biol. Chem..

[bib0022] Ghasemi H.A., Shivazad M., Mirzapour Rezaei S.S., Karimi Torshizi M.A. (2016). Effect of synbiotic supplementation and dietary fat sources on broiler performance, serum lipids, muscle fatty acid profile and meat quality. Br. Poult. Sci..

[bib0023] Ghazanfari S., Shiri Ghzghapan A., Honarbakhsh S. (2024). Effects of peppermint essential oil and artifier on growth performance, carcass characteristics and nutrient digestibilities in broiler chickens fed with low energy diets. Vet. Anim. Sci..

[bib0024] Gholami M., Shirzadi H., Taherpour K., Rahmatnejad E., Shokri A., Khatibjoo A. (2024). Effect of emulsifier on growth performance, nutrient digestibility, intestinal morphology, faecal microbiology and blood biochemistry of broiler chickens fed low-energy diets. Vet. Med. Sci..

[bib0025] Grela E.R., Świątkiewicz M., Florek M., Bąkowski M., Skiba G. (2021). Effect of inulin source and a probiotic supplement in pig diets on carcass traits, meat quality and fatty acid composition in finishing pigs. Animals.

[bib0026] Haetinger V.S., Dalmoro Y.K., Godoy G.L., Lang M.B., de Souza O.F., Aristimunha P., Stefanello C. (2021). Optimizing cost, growth performance, and nutrient absorption with a bio-emulsifier based on lysophospholipids for broiler chickens. Poult. Sci..

[bib0027] Heshmati J. (2021). Effect of omega-3 fatty acid supplementation on gene expression of inflammation, oxidative stress and cardiometabolic parameters: systematic review and meta-analysis. J. Funct. Foods.

[bib0028] Huang S., Dong S., Lin L., Ma Q., Xu M., Ni L., Fan Q. (2023). Inulin ameliorates metabolic syndrome in high-fat diet-fed mice by regulating gut microbiota and bile acid excretion. Front. Pharmacol..

[bib0029] Jang A., Liu X.-D., Shin M.-H., Lee B.-D., Lee S.-K., Lee J.-H., Jo C. (2008). Antioxidative potential of raw breast meat from broiler chicks fed a dietary medicinal herb extract mix. Poult. Sci..

[bib85] Junge W., Leybold K., Kraack B. (1983). Influence of colipase on the turbidimetric determination of pancreatic lipase catalytic activity. Clin. Chem. Lab. Med..

[bib0030] Kanakri K., Carragher J., Hughes R., Muhlhausler B., Gibson R. (2018). The effect of different dietary fats on the fatty acid composition of several tissues in broiler chickens. Eur. J. Lipid Sci. Technol..

[bib0031] Kareem K.Y., Loh T.C., Foo H.L., Akit H., Samsudin A.A. (2016). Effects of dietary postbiotic and inulin on growth performance, IGF1 and GHR mRNA expression, faecal microbiota and volatile fatty acids in broilers. BMC Vet. Res..

[bib0032] Khajali F., Fahimi S. (2010). Influence of dietary fat source and supplementary α-tocopheryl acetate on pulmonary hypertension and lipid peroxidation in broilers. J. Anim. Physiol. Anim. Nutr..

[bib0033] Khajeh Bami M., Afsharmanesh M., Espahbodi M., Esmaeilzadeh E. (2022). Effects of dietary nano-selenium supplementation on broiler chicken performance, meat selenium content, intestinal microflora, intestinal morphology, and immune response. J. Trace Elem. Med. Biol..

[bib0034] Khonyoung D., Yamauchi K., Suzuki K. (2015). Influence of dietary fat sources and lysolecithin on growth performance, visceral organ size, and histological intestinal alteration in broiler chickens. Livest. Sci..

[bib0035] Ko H., Wang J., Chiu J.W.-C., Kim W.K. (2023). Effects of metabolizable energy and emulsifier supplementation on growth performance, nutrient digestibility, body composition, and carcass yield in broilers. Poult. Sci..

[bib0036] Kozlowska I., Marc-Pienkowska J., Bednarczyk M. (2016). Beneficial aspects of inulin supplementation as a fructooligosaccharide prebiotic in monogastric animal nutrition—A review. Ann. Anim. Sci..

[bib0037] Liu J.B., Cao S.C., Liu J., Xie Y.N., Zhang H.F. (2018). Effect of probiotics and xylo-oligosaccharide supplementation on nutrient digestibility, intestinal health and noxious gas emission in weanling pigs. Asian-Australas. J. Anim. Sci..

[bib0038] Liu N., Deng X., Wang J., Dong S. (2023). Effect of lysophospholipids on the growth performance, nutrient digestibility, lipid metabolism and meat quality of fattening rabbits. Arch. Anim. Nutr..

[bib0039] Luo D., Li J., Xing T., Zhang L., Gao F. (2021). Combined effects of xylo-oligosaccharides and coated sodium butyrate on growth performance, immune function, and intestinal physical barrier function of broilers. Anim. Sci. J..

[bib0040] Lynn K.R., Clevette-Radford N.A. (1984). Purification and characterization of hevain, a serine protease from *Hevea brasiliensis*. Phytochemistry.

[bib0041] Majdolhosseini L., Ghasemi H.A., Hajkhodadadi I., Moradi M.H. (2019). Nutritional and physiological responses of broiler chickens to dietary supplementation with de-oiled soybean lecithin at different metabolisable energy levels and various fat sources. Br. J. Nutr..

[bib0042] Matarneh S.K., Yen C.-N., Elgin J.M., Beline M., da Luz e Silva S., Wicks J.C., England E.M., Dalloul R.A., Persia M.E., Omara I.I., Shi H., Gerrard D.E. (2018). Phosphofructokinase and mitochondria partially explain the high ultimate pH of broiler *pectoralis major* muscle. Poult. Sci..

[bib0043] Michels D., Verkempinck S.H.E., Vermeulen K., Spaepen R., Burton E., Scholey D., Wealleans A.L., Grauwet T. (2025). An innovative approach to emulsifier use in broiler feed affects nutrient digestion and growth performance in young broilers. Br. Poult. Sci..

[bib0044] Mohiti-Asli M., Ghanaatparast-Rashti M., Sharifi S.D., Rouhanipour H., Akbarian P. (2025). Effects of lysophospholipid supplementation in diets with different oil sources on broiler performance under heat stress. Front. Vet. Sci..

[bib0045] Moreno-Mendoza Y., López-Villarreal K.D., Hernández-Martínez C.A., Rodríguez-Tovar L.E., Hernández-Coronado A.C., Soto-Domínguez A., Hume M.E., Méndez-Zamora G. (2021). Effect of moringa leaf powder and agave inulin on performance, intestinal morphology, and meat yield of broiler chickens. Poult. Sci..

[bib0046] Mueller W. (1956). Feasibility of the chromic oxide and lignin indicator methods for metabolism experiments with chickens. J. Nutr..

[bib0047] Narala V.R., Jugbarde M.A., Orlovs I., Masin M. (2022). Inulin as a prebiotic for the growth of vegan yoghurt culture in pea protein-based vegan yoghurt-ice cream, while improving the textural properties. Appl. Food Res..

[bib0048] Nari N., Ghasemi H.A. (2020). Growth performance, nutrient digestibility, bone mineralization, and hormone profile in broilers fed phosphorus-deficient diets supplemented with butyric acid and *Saccharomyces boulardii*. Poult. Sci..

[bib0049] Obianwuna U.E., Qiu K., Wang J., Zhang H., Qi G., Huang L., Wu S. (2023). Effects of dietary *Clostridium butyricum* and fructooligosaccharides, alone or in combination, on performance, egg quality, amino acid digestibility, jejunal morphology, immune function, and antioxidant capacity of laying hens. Front. Microbiol..

[bib0050] Oketch E.O., Wickramasuriya S.S., Oh S., Choi J.S., Heo J.M. (2023). Physiology of lipid digestion and absorption in poultry: an updated review on the supplementation of exogenous emulsifiers in broiler diets. J. Anim. Physiol. Anim. Nutr..

[bib0051] Okur N. (2020). The effects of soy oil, poultry fat and tallow with fixed energy:protein ratio on broiler performance. Arch. Anim. Breed..

[bib0052] Özlüer Hunt A., Çetinkaya M., Özkan Yılmaz F., Yıldırım M., Berkoz M., Yalın S. (2019). Effect of dietary supplementation of inulin on growth performance, digestion enzyme activities and antioxidant status of rainbow trout (*Oncorhynchus mykiss*). Turk. J. Agric. Food Sci. Technol..

[bib0053] Pashaei Jalal M., Sharifi S.D., Honarbakhsh S., Rouhanipour H. (2024). Effects of low energy diets supplemented with emulsifier on growth performance, nutrient digestibility, and intestinal morphology of broiler chickens. Livest. Sci..

[bib0054] Pereira G.G., Garcia R.K.A., Ferreira L.L., Barrera-Arellano D. (2017). Soybean and soybean/beef-tallow biodiesel: A comparative study on oxidative degradation during long-term storage. J. Am. Oil Chem. Soc..

[bib0055] Pizones Ruiz-Henestrosa V.M., Ribourg L., Kermarrec A., Anton M., Pilosof A., Viau M., Meynier A. (2022). Emulsifiers modulate the extent of gastric lipolysis during the dynamic *in vitro* digestion of submicron chia oil/water emulsions with limited impact on the final extent of intestinal lipolysis. Food Hydrocoll.

[bib0056] Poorghasemi M., Chamani M., Mirhosseini S.Z., Saravy A., Sadeghi A.-A., Seidavi A. (2024). Effects of different lipid sources with or without a probiotic on gastrointestinal tract, immune system and blood parameters of chickens: an animal model. Lipids.

[bib0057] Rodríguez-Sánchez R., Tres A., Sala R., Guardiola F., Barroeta A.C. (2019). Evolution of lipid classes and fatty acid digestibility along the gastrointestinal tract of broiler chickens fed different fat sources at different ages. Poult. Sci..

[bib0058] Saleh A.A., Alharthi A.S., Alhotan R.A., Atta M.S., Abdel-Moneim A.-M.E. (2021). Soybean oil replacement by poultry fat in broiler diets: performance, nutrient digestibility, plasma lipid profile and muscle fatty acids content. Animals.

[bib0059] Sanz M. (1999). Higher lipid accumulation in broilers fed on saturated fats than in those fed on unsaturated fats. Br. Poult. Sci..

[bib0060] Shahid I., Sharif M.I., Yousaf M.I., Ahmad F.I., Virk M.R., Bilal M.Q., Anwar U., Ali A., Hussain M.I., Chishti M.F.A., Rahman M.A. (2021). Effect of exogenous emulsifier (lyso-phospholipid) supplementation in the broiler diet on feed intake and growth performance during grower phase. Braz. J. Poult. Sci..

[bib0061] Shang H.-M., Zhou H.-Z., Yang J.-Y., Li R., Song H., Wu H.-X. (2018). *In vitro* and *in vivo* antioxidant activities of inulin. PLoS One.

[bib0062] Shoaib M., Bhatti S.A., Ashraf S., Hamid M.M.A., Najam-us-Sahar M.M.Javed, Amir S., Aslam N., Roobi A., Iqbal H.H., Asif M.A., Nazir U., Saif-ur-Rehman M. (2023). Fat digestion and metabolism: effect of different fat sources and fat mobilisers in broilers’ diet on growth performance and physiological parameters—A review. Ann. Anim. Sci..

[bib0063] Shoaib M., Bhatti S.A., Iqbal H., Ashraf S., Najam-us-Sahar M.M.A.Hamid, Asim M., Hussain M. (2022). Production efficiency, nutrient digestibility, and meat quality of broilers reared on different fat sources and emulsifiers. Turk. J. Vet. Anim. Sci..

[bib0064] Siyal F.A., Babazadeh D., Wang C., Arain M.A., Saeed M., Ayasan T., Zhang L., Wang T. (2017). Emulsifiers in the poultry industry. World’s Poult. Sci. J..

[bib0065] Somogyi M. (1960). Modification of two methods for the assay of amylase. Clin. Chem..

[bib0066] Song J., Li Q., Li P., Liu R., Cui H., Zheng M., Everaert N., Zhao G., Wen J. (2018). The effects of inulin on the mucosal morphology and immune status of specific pathogen-free chickens. Poult. Sci..

[bib0067] Tang R., Chen J., Liang S., Zhao J., Chen B., Xu Y., Ding X., Fu A., Zhan X. (2025). Effects of *Lactobacillus reuteri* and its combination with gluco-oligosaccharides on improving growth performance and intestinal health of broilers. Anim. Nutriomics.

[bib0068] Teng P.-Y., Adhikari R., Llamas-Moya S., Kim W.K. (2021). Effects of combination of mannan-oligosaccharides and β-glucan on growth performance, intestinal morphology, and immune gene expression in broiler chickens. Poult. Sci..

[bib0069] Tenório K.I., Eyng C., do Amaral Duarte C.R., Vianna Nunes R., Broch J., Rohloff Júnior N., Köhler T.L., Hagdon Cirilo E. (2022). Effect of lipid source and emulsifier on productive and physiological parameters of broilers. Anim. Biosci..

[bib0070] Vakili R., Ebrahimnezhad Y. (2023). Impact of dietary supplementation of unsaturated and saturated fatty acids on bone strength, fatty acids profile of thigh muscle and immune responses in broiler chickens under heat stress. Vet. Med. Sci..

[bib0071] Wang Y., Zeng D., Wei L., Chen J., Li H., Wen L., Huang G., Dai Z., Luo J., Sun J., Xi Q., Zhang Y., Chen T. (2024). Effects of emulsifiers on lipid metabolism and performance of yellow-feathered broilers. BMC Vet. Res..

[bib84] Wickramasuriya S.S., Macelline S.P., Cho H.M., Hong J.S., Park S.H., Heo J.M. (2020). Physiological effects of a tallow-incorporated diet supplemented with an emulsifier and microbial lipases on broiler chickens. Front. Vet. Sci..

[bib0072] Wirnt R., Bergmeyer H.U. (1974). Methods of enzymatic analysis.

[bib0073] Wirnt R., Bergmeyer H.U. (1974). Methods of enzymatic analysis.

[bib0074] Wongsuthavas S., Yuangklang C., Vasupen K., Mitchaothai J., Alhaidary A., Mohamed H.E., Beynen A.C. (2011). Fatty acid and energy metabolism in broiler chickens fed diets containing either beef tallow or an oil blend. J. Anim. Physiol. Anim. Nutr..

[bib0075] Wu X.Z., Wen Z.G., Hua J.L. (2019). Effects of dietary inclusion of *Lactobacillus* and inulin on growth performance, gut microbiota, nutrient utilization, and immune parameters in broilers. Poult. Sci..

[bib0076] Xia Y., Miao J., Zhang Y., Zhang H., Kong L., Seviour R. (2021). Dietary inulin supplementation modulates the composition and activities of carbohydrate-metabolizing organisms in the cecal microbiota of broiler chickens. PLoS One.

[bib0077] Xiang S., Zhao M., Li Z., Zheng H., Ding Y., Peng F., Xiao J., Xie K., Zhang H., He X., Song Z. (2024). Effects of lysolecithin on growth performance, nutrient digestibility and liver lipid metabolism of yellow-feather broilers. Ital. J. Anim. Sci..

[bib0078] Yan Z., Tang X., Wu R., Yang C., Jiang Y., Wang X., Tang Q., Hu Y., Wang L., Jiang Z. (2025). Effect of fructo-oligosaccharides on growth performance and meat quality in broilers. Front. Vet. Sci..

[bib0079] Yang X., Zhang M., Liu Y., Wei F., Li X., Feng Y., Jin X., Liu D., Guo Y., Hu Y. (2023). Inulin-enriched *Megamonas funiformis* ameliorates metabolic dysfunction-associated fatty liver disease by producing propionic acid. NP J. Biofilms Microbiom..

[bib0080] Yang J., Qin K., Sun Y., Yang X. (2024). Microbiota-accessible fiber activates short-chain fatty acid and bile acid metabolism to improve intestinal mucus barrier in broiler chickens. Microbiol. Spectr..

[bib0081] Ye X., Yu Y., Chen J., Zou Y., Liu S., Tan H., Zhao F., Wang Y. (2022). Evaluation of lipid sources and emulsifier addition on fat digestion of yellow-feathered broilers. J. Anim. Sci..

[bib0082] Yousefi J., Taherpour K., Ghasemi H.A., Akbari Gharaei M., Mohammadi Y., Rostami F. (2023). Effects of emulsifier, betaine, and L-carnitine on growth performance, immune response, gut morphology, and nutrient digestibility in broiler chickens exposed to cyclic heat stress. Br. Poult. Sci..

[bib0083] Zhang M., Wang S., Qin C., Li M. (2023). Dietary inulin benefits on growth, digestive ability and intestinal microbiota in yellow catfish exposed to ammonia. Aquac. Rep..

