# Supplementary material for: Fat source–dependent effects of lysophospholipid and inulin supplementation in broilers: Impacts on performance, muscle fatty acids, digestibility, enzyme activity, and intestinal morphology
Source: Poult Sci. 2026 Jan 28;105(4):106531. doi: 10.1016/j.psj.2026.106531 (PMC12892074; doi:10.1016/j.psj.2026.106531)
Supplement: Supplementary file 1 [file mmc1.docx]

**Table S1**. Effects of fat source [soybean oil (SO) and beef tallow (BT)] and dietary supplementation with a lysophospholipid blend (LPL) and inulin on mean composition of saturated fatty acids (SFA), monounsaturated fatty acids (MUFA), and polyunsaturated fatty acids (PUFA) of breast muscle in broiler chickens at 42 days of age

|  |  |  | SFA (%) | | |  | MUFA (%) | | |  | PUFA (%) | | | | |
| --- | --- | --- | --- | --- | --- | --- | --- | --- | --- | --- | --- | --- | --- | --- | --- |
| Fat source | EB (g/kg) | Inulin (g/kg) | C14:0 | C16:0 | C18:0 |  | C16:1 | C18:1 | C20:1 |  | C18:2 | C20:4 | C18:3 | C20:5 | C22:6 |
| SO | - | - | 1.11 | 20.18 | 6.12 |  | 2.65 | 32.60 | 0.212 |  | 33.69 | 1.64 | 1.51 | 0.097 | 0.208 |
| SO | - | + | 0.95 | 19.79 | 6.06 |  | 2.64 | 31.97 | 0.237 |  | 34.75 | 1.68 | 1.61 | 0.116 | 0.195 |
| SO | + | - | 1.03 | 19.80 | 6.20 |  | 2.48 | 30.23 | 0.222 |  | 36.34 | 1.66 | 1.69 | 0.115 | 0.232 |
| SO | + | + | 0.89 | 19.30 | 5.33 |  | 2.82 | 31.35 | 0.269 |  | 36.60 | 1.25 | 1.79 | 0.135 | 0.269 |
| BT | - | - | 1.34 | 23.51 | 7.03 |  | 2.66 | 36.04 | 0.572 |  | 26.24 | 1.23 | 1.17 | 0.087 | 0.123 |
| BT | - | + | 1.22 | 22.62 | 6.72 |  | 2.64 | 35.89 | 0.454 |  | 27.76 | 1.37 | 1.13 | 0.072 | 0.113 |
| BT | + | - | 1.39 | 22.98 | 7.40 |  | 2.61 | 33.93 | 0.431 |  | 27.82 | 1.76 | 1.37 | 0.108 | 0.207 |
| BT | + | + | 1.23 | 21.84 | 6.96 |  | 2.69 | 34.32 | 0.383 |  | 29.51 | 1.45 | 1.34 | 0.104 | 0.165 |
| SEM | | | 0.057 | 0.378 | 0.189 |  | 0.173 | 1.055 | 0.0439 |  | 1.113 | 0.162 | 0.126 | 0.0211 | 0.0278 |
| Main effect means | | |  |  |  |  |  |  |  |  |  |  |  |  |  |
| Fat source | | |  |  |  |  |  |  |  |  |  |  |  |  |  |
| SO | | | 0.99^b^ | 19.77^b^ | 5.93^b^ |  | 2.65 | 31.54^b^ | 0.235^b^ |  | 35.34^a^ | 1.56 | 1.65^a^ | 0.116 | 0.226^a^ |
| BT | | | 1.30^a^ | 22.74^a^ | 7.03^a^ |  | 2.65 | 35.05^a^ | 0.460^a^ |  | 27.83^b^ | 1.45 | 1.25^b^ | 0.093 | 0.152^b^ |
| SEM | | | 0.028 | 0.189 | 0.094 |  | 0.087 | 0.527 | 0.0220 |  | 0.557 | 0.081 | 0.063 | 0.0105 | 0.0139 |
| EB (g/kg) | | |  |  |  |  |  |  |  |  |  |  |  |  |  |
| - | | | 1.16 | 21.53^a^ | 6.48 |  | 2.64 | 34.12^a^ | 0.369 |  | 30.61^b^ | 1.48 | 1.36^b^ | 0.093 | 0.160^b^ |
| + | | | 1.14 | 20.98^b^ | 6.47 |  | 2.65 | 32.46^b^ | 0.326 |  | 32.57^a^ | 1.53 | 1.55^a^ | 0.115 | 0.218^a^ |
| SEM | | | 0.028 | 0.189 | 0.094 |  | 0.087 | 0.527 | 0.0220 |  | 0.557 | 0.081 | 0.063 | 0.0105 | 0.0139 |
| Inulin (g/kg) | | |  |  |  |  |  |  |  |  |  |  |  |  |  |
| - | | | 1.22^a^ | 21.62^a^ | 6.69^a^ |  | 2.60 | 33.20 | 0.359 |  | 31.02 | 1.57 | 1.43 | 0.102 | 0.192 |
| + | | | 1.07^b^ | 20.89^b^ | 6.27^b^ |  | 2.70 | 33.38 | 0.336 |  | 32.16 | 1.44 | 1.47 | 0.106 | 0.185 |
| SEM | | | 0.028 | 0.189 | 0.094 |  | 0.087 | 0.527 | 0.0220 |  | 0.557 | 0.081 | 0.063 | 0.0105 | 0.0139 |
| Significance | | |  |  |  |  |  |  |  |  |  |  |  |  |  |
| Fat source | | | <0.001 | <0.001 | <0.001 |  | 0.974 | <0.001 | <0.001 |  | <0.001 | 0.372 | <0.001 | 0.1327 | 0.001 |
| EB | | | 0.640 | 0.049 | 0.938 |  | 0.948 | 0.033 | 0.171 |  | 0.018 | 0.641 | 0.042 | 0.150 | 0.005 |
| inulin | | | 0.001 | 0.011 | 0.004 |  | 0.434 | 0.809 | 0.436 |  | 0.160 | 0.255 | 0.719 | 0.740 | 0.650 |
| Fat × EB | | | 0.232 | 0.681 | 0.024 |  | 0.987 | 0.817 | 0.053 |  | 0.712 | 0.036 | 0.898 | 0.740 | 0.614 |
| Fat × inulin | | | 0.902 | 0.290 | 0.708 |  | 0.610 | 0.932 | 0.073 |  | 0.553 | 0.660 | 0.456 | 0.355 | 0.316 |
| EB × inulin | | | 0.882 | 0.750 | 0.089 |  | 0.371 | 0.447 | 0.455 |  | 0.839 | 0.059 | 0.969 | 0.842 | 0.801 |
| Fat × EB × inulin | | | 0.768 | 0.900 | 0.208 |  | 0.610 | 0.686 | 0.690 |  | 0.760 | 0.993 | 0.943 | 1.000 | 0.316 |

^a,b^ Means within each column with no common superscript differ (*P* < 0.05).
